# Clinicopathological Significance of Cell Adhesion Molecule 4 Expression in Gallbladder Cancer and Its Prognostic Role

**DOI:** 10.3390/ijms24086898

**Published:** 2023-04-07

**Authors:** Seongsik Bang, Seungyun Jee, Hwangkyu Son, Hyebin Cha, Kihyuk Song, Hosub Park, Jaekyung Myung, Hyunsung Kim, Seungsam Paik

**Affiliations:** Department of Pathology, Seoul Hospital, Hanyang University College of Medicine, Seoul 04763, Republic of Korea; grypony@naver.com (S.B.); jee.seung.yun@gmail.com (S.J.); ganzi4900@gmail.com (H.S.); chbin0111@gmail.com (H.C.); kihyuk1127@naver.com (K.S.); parkhstm@gmail.com (H.P.); tontos016@naver.com (J.M.)

**Keywords:** gallbladder cancer, prognosis, immunohistochemistry, cell adhesion molecule 4, CADM4

## Abstract

Cell adhesion molecule 4 (CADM4) is involved in intercellular interactions and is a tumor-suppressor candidate. The role of CADM4 in gallbladder cancer (GBC) has not been reported. Therefore, the clinicopathological significance and prognostic value of CADM4 expression in GBC were evaluated in the present study. Immunohistochemistry (IHC) was performed on 100 GBC tissues to assess CADM4 expression at the protein level. The association between CADM4 expression and the clinicopathological characteristics of GBC was analyzed, and the prognostic significance of CADM4 expression was evaluated. Low CADM4 expression was significantly associated with advanced T category (*p* = 0.010) and high AJCC stage (*p* = 0.019). In a survival analysis, low CADM4 expression was associated with shorter overall survival (OS; *p* = 0.001) and recurrence-free survival (RFS; *p* = 0.018). In univariate analyses, low CADM4 expression was associated with shorter OS (*p* = 0.002) and RFS (*p* = 0.023). In multivariate analyses, low CADM4 expression was an independent prognostic factor of OS (*p* = 0.013). Low CADM4 expression was associated with tumor invasiveness and poor clinical outcomes in patients with GBC. CADM4 may play an important role in cancer progression and patient survival and can be used as a potential prognostic marker of GBC.

## 1. Introduction

Gallbladder cancer (GBC) is a relatively rare malignancy, accounting for 0.6% of new cancer cases and 0.9% of all cancer-related deaths in 2020, according to global cancer statistics [1]. However, GBC is the most common cancer originating in the biliary tract, and it is often detected at an advanced stage in patients and has a poor prognosis [2]. GBC is prevalent in women and is associated with several risk factors such as gallstone disease, chronic infection, primary sclerosing cholangitis, and choledochal cysts. With a few exceptions, the incidence is declining in the Western world due to cholecystectomy [3].

The only curative treatment for GBC is complete surgical resection, but only approximately 10% of patients with early-stage disease are candidates for the procedure [4]. Most GBC patients are diagnosed in advanced stages and show poor prognosis despite conventional chemotherapy [5]. Therefore, the discovery of prognostic biomarkers and potential therapeutic targets based on the molecular background of GBC is needed. Based on the signaling pathway and genetic alteration identified in GBC, novel therapeutic agents, including mTOR inhibitors, tyrosine kinase inhibitors (e.g., VEGF, HER2, EGFR), and MAPK/ERK kinases inhibitors, are undergoing a clinical trial [5]. Serum tumor markers (CEA, CA 19-9, CA 242), tyrosine kinase receptors (VEGF-A, ER1, HDGF, HER2), and several other potential molecules (e.g., survivin, PTEN, SHP2, EpCAM) are prognostic biomarkers that affect the survival of GBC patients [6].

Cell adhesion molecule 4 (CADM4), also called TSLL2, IGSF4C, SynCAM4, or Necl-4, is a membrane protein that belongs to the immunoglobulin (Ig) superfamily and is involved in cell–cell interactions, including intercellular adhesion [7,8]. CADM4 is expressed in human tissues such as neural (brain, spinal cord), urinary (kidney, bladder, prostate), and spleen [8]. Loss of or reduced CADM4 expression has been found in cancer cells, including glioma, prostate cancer, renal cell carcinoma, and non-small cell lung cancer; thus, CADM4 has been recognized as a candidate tumor suppressor [7,9,10].

Despite the discovery of many candidates, no prognostic biomarkers are yet ready for clinical use in GBC. The expression of CADM4 can be assessed using immunohistochemistry (IHC) at the protein level and has shown an association with prognosis [11]. Accordingly, CADM4 is a biomarker candidate for cost-effectively predicting the prognosis of patients in daily practice. The CADM4 expression in GBC and its clinicopathological significance have not yet been reported. Therefore, the role of CADM4 in GBC was investigated and its potential as a prognostic biomarker evaluated in the present study. IHC was performed on human GBC tissues to evaluate the CADM4 expression at the protein level and to analyze the association with clinicopathological characteristics. In addition, we investigated biological pathways involving CADM4-related gene sets using online bioinformatics tools.

## 2. Results

### 2.1. Clinicopathological Characteristics of Patients

The median age of the patients was 65 years (range: 28–90 years). The cohort included 51 female and 49 male patients. The majority of GBC originated from the gallbladder body (48%), fundus (22%), and neck (12%). Most of the cases were adenocarcinoma (96%) and a few cases were classified as adenosquamous carcinoma. Lymphovascular invasion was identified in 50 cases (50.0%) and perineural invasion in 32 cases (32.0%). Lymph node metastases were found in 36 cases (36.0%) and the majority of patients (83%) were classified as above stage I, according to the 8th AJCC staging system. Using the SISH technique, nine HER2 amplification cases (9%) were identified. The clinicopathological characteristics of patients with GBC are summarized in Table 1.

### 2.2. Correlations between CADM4 Expression and Clinicopathological Characteristics

The low CADM4 expression group (IRS ≤ 4) consisted of 28 cases. Low CADM4 expression was significantly associated with advanced T category (*p* = 0.010) and high AJCC stage (*p* = 0.019). Other clinicopathological characteristics, including age, sex, histological grade, lymphovascular invasion, perineural invasion, lymph node metastasis, and HER2 status, were not significantly associated with CADM4 expression. The correlations between CADM4 expression and clinicopathological characteristics are summarized in Table 2.

### 2.3. Prognostic Implication of CADM4 Expression

**Figure 1:**
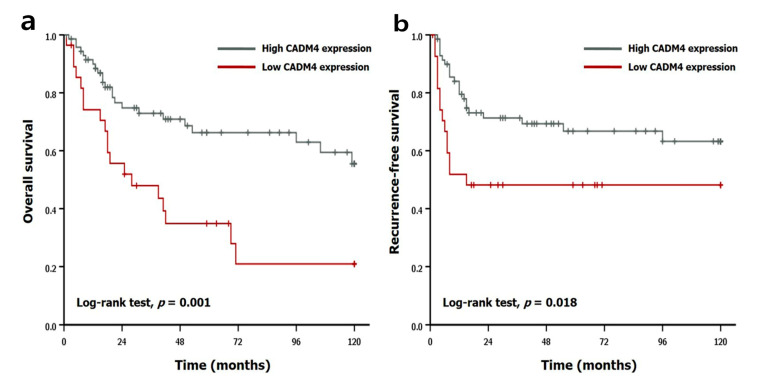
Survival analyses using Kaplan–Meier method in patients with gallbladder cancer (GBC). (**a**) Overall survival (OS; log-rank test, *p*-value: 0.001). (**b**) Recurrence-free survival (RFS; log-rank test, *p*-value: 0.018).

Patients with low CADM4 expression showed significantly shorter OS and RFS compared with subjects with high CADM4 expression (*p* = 0.001 and *p* = 0.018, respectively; Figure 1) in a Kaplan–Meier survival analysis. In univariate analyses, low CADM4 expression (*p* = 0.002), advanced T category (*p* = 0.001), lymph node metastasis (*p* = 0.002), high AJCC stage (*p* < 0.001), lymphovascular invasion (*p* = 0.004), and perineural invasion (*p* = 0.004) were predictors of short OS. In addition, low CADM4 expression (*p* = 0.023), advanced T category (*p* < 0.001), lymph node metastasis (*p* = 0.001), high AJCC stage (*p* < 0.001), lymphovascular invasion (*p* < 0.001), and perineural invasion (*p* = 0.001) were predictors of short RFS. Multivariate analyses for OS revealed that low CADM4 expression (*p* = 0.013) and high AJCC stage (*p* = 0.040) were independent prognostic factors. In multivariate analyses for RFS, short RFS was significantly associated with high AJCC stage (*p* = 0.001); however, low CADM4 expression was not statistically significant (*p* = 0.058). HER2 amplification did not show a significant association with the prognosis of GBC patients. The results of the univariate and multivariate analyses are shown in Table 3.

### 2.4. Gene Ontology (GO) and Kyoto Encyclopedia of Genes and Genomes (KEGG) Pathways

**Figure 2:**
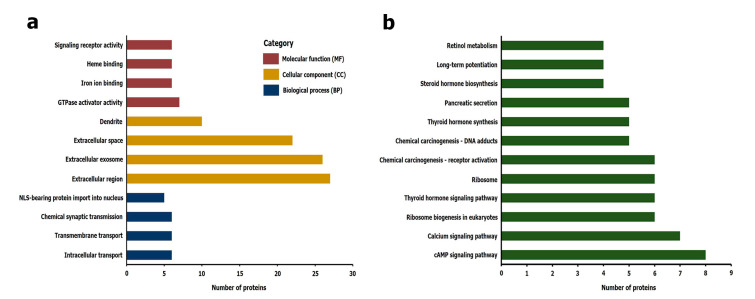
Representative list of the significantly enriched (**a**) Gene Ontology (GO) terms and Kyoto Encyclopedia of Genes and (**b**) Genomes (KEGG) pathways.

GO and KEGG pathway analyses were executed using the DAVID database for 212 targets. GO divided the biological domain into three categories: molecular function (MF), cellular component (CC), and biological process (BP). We evaluated the detailed functions involved in the target gene. By analyzing the MF category, we found that the target genes were involved in GTPase activator activity, iron ion binding, heme binding, signaling receptor activity, etc. Extracellular domains, extracellular exosomes, extracellular space, and dendrites accounted for a high proportion of the CC category. The BP category was dominated by intracellular transport, transmembrane transport, chemical synaptic transmission, and NLS-bearing protein import into the nucleus. The KEGG pathway analysis showed that the target genes were associated with functional pathways such as the cAMP signaling pathway, calcium signaling pathway, and ribosome biogenesis in eukaryotes. The representative results in the GO and KEGG pathway analyses are presented in Figure 2, and all significant pathways are summarized in Table S1.

## 3. Discussion

In the present study, CADM4 expression and its clinicopathological significance in GBC were investigated using IHC staining at the protein level. Low CADM4 expression was found in 28 GBC cases, which showed a significant association with advanced T category (*p* = 0.010) and high AJCC stage (*p* = 0.019). In survival analyses, patients with low CADM4 expression showed shorter OS and RFS than subjects with high CADM4 expression (*p* = 0.001 and *p* = 0.018, respectively). Low CADM4 expression was an independent prognostic factor affecting OS (*p* = 0.013) based on Cox regression analysis. In addition, we revealed 53 pathways involving CADM4-related gene sets using GO and KEGG pathway analyses.

The role of CADM4 in tumor growth and invasion has been studied in several types of tumors. Nagata et al. showed that CADM4 inhibits tumor formation in a cell line of renal cell carcinoma [9]. In HCC cells, Suresh et al. revealed that CADM4 overexpression reduced tumor formation [12]. Luo et al. reported that CADM4 knockdown promotes tumor growth in non-small cell lung cancer [10], and Raveh et al. suggested that CADM4 inhibits tumor growth formed by colon cancer cells [13]. Saito et al. and Kim et al. reported that loss of or low CADM4 expression was associated with an advanced stage of breast cancer [14] and small intestinal adenocarcinoma [11], respectively. These reports support the role of CADM4 as a potential tumor suppressor.

**Figure 3:**
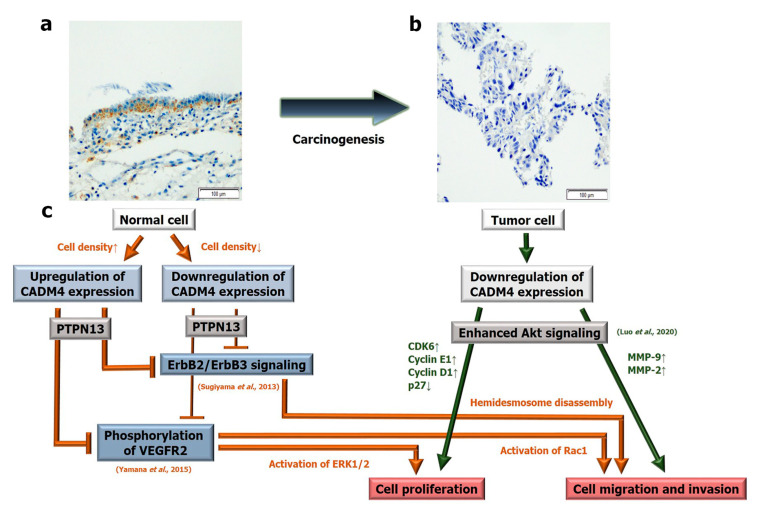
(**a**) Normal cells of the gallbladder mucosa showed relatively uniform expression of CADM4 with weak to moderate intensity (×200). (**b**) Loss of or reduced CADM4 expression was identified in gallbladder cancer (GBC) tissues (×200). (**c**) Schematic representation of mechanisms by which regulation of CADM4 expression in normal and tumor cells contributes to cell proliferation, migration, and invasion.

The exact mechanism by which CADM4 regulates tumors is unknown; however, studies have revealed the interaction of CADM4 with other surface molecules in extracellular regions. Yamana et al. suggested that the interaction between CADM4 and VEGF receptors regulates contact inhibition by inhibiting tyrosine phosphorylation of VEGF receptors through protein-tyrosine phosphatase, non-receptor type 13 (PTPN13) [15]. Sugiyama et al. suggested that CADM4 inhibits cell movement and survival by interacting with PTPN13 and inhibiting ErbB2/ErbB3 signaling. The authors also identified that the downregulation of CADM4 in tumor cells promotes hemidesmosome disassembly, leading to tumor cell invasion and metastasis [16]. Figure 3 shows the expression pattern and biological pathways of CADM4 in normal and tumor cells.

HER2 amplification (or overexpression) in GBC has been investigated in several studies. HER2 status was assessed using IHC with in situ hybridization, and HER2 positivity was shown in 10.4–13.9% of cases [17,18,19]. A significant association was not found between HER2 positivity and survival rate. In the present study, HER2 amplification was confirmed in nine cases (9%), and its effect on clinical outcomes could not be identified. In a previous study of gastric cancer, low CADM4 expression was more frequently observed in tumors without HER2 amplification [20]. However, Saito et al. did not identify the association between HER2 status and CADM4 expression in breast cancer [14]; in the present GBC study, significant results could also not be confirmed.

There were several limitations to our study. This was a retrospective study, with a limited number of cases from a single institution. The expression of CADM4 in human GBC tissues was assessed using immunohistochemistry alone. Therefore, the biological pathway associated with the expression of CADM4 could not be explained. Further bioinformatic analysis and experimental studies on the regulatory mechanisms of CADM4 expression and specific downstream targets of the CADM4 protein are required to determine the exact role of CADM4.

In conclusion, CADM4 expression in GBC at the protein level was identified using IHC. This study is the first in which CADM4 expression in human GBC tissues was evaluated and, similar to other human cancer studies, the results indicated that CADM4 may play a crucial role in GBC as a tumor suppressor and prognostic biomarker.

## 4. Materials and Methods

### 4.1. Patients and Specimens

This study included GBC patients who underwent curative surgery at Hanyang University Hospital (Seoul, Republic of Korea) from January 1991 to September 2018. A total of 124 GBC cases were retrospectively enrolled; 2 cases with missing follow-up and 16 without sufficient tumor tissue were excluded from the study. In addition, 6 cases were excluded due to the possibility of death caused by surgical complications (death within 1 month after surgery). Among patients with tumor invasion in the main portal vein or hepatic artery (pT4), 3 cases in whom clean resection margins were obtained through additional combined resection (hepatic artery and portal vein) were included in this study [21]. An additional 8 samples of normal mucosa were obtained to assess CADM4 expression in normal cells. This study was approved by the Institutional Review Board of Hanyang University Hospital (IRB file No. HYUH 2018-08-031-002), and the requirement for informed consent was waived.

### 4.2. Collection of Clinicopathological Data

The following clinical characteristics were obtained using the electronic medical records: patient age, sex, follow-up period, survival status, and recurrence status. All hematoxylin and eosin (H&E)-stained slides used at the time of diagnosis and pathology reports were reviewed to determine the pathological characteristics, including tumor size, tumor site, histologic type, histologic grade, pathologic stage, lymphovascular invasion, and perineural invasion. Pathologic stage was assessed using the 8th edition of the American Joint Committee on Cancer (AJCC).

### 4.3. Tissue Microarray (TMA) Construction and Immunohistochemistry

To perform immunohistochemistry (IHC) efficiently on a large number of cases, tissue microarrays (TMAs) were constructed [22]. First, a representative portion of the tumor from H&E-stained sections was marked under light microscopy. Then, a cylindrical tissue core (3.0 mm in diameter) was obtained from the corresponding formalin-fixed, paraffin-embedded tissue blocks (donor block) and inserted into empty recipient blocks (Unitma, Gyeonggi-do, Korea). Each TMA block consisted of 6 × 5 tumor samples.

IHC staining for CADM4 was performed on 4 μm thick sections from the TMA blocks using the Benchmark XT automated staining system (Ventana Medical Systems, Tucson, AZ, USA), according to the manufacturer’s protocol. A recombinant rabbit anti-CADM4 antibody (SAB4500746, Sigma-Aldrich, St. Louis, MO, USA; diluted 1:200) was used as the primary antibody.

### 4.4. Interpretation of IHC

**Figure 4:**
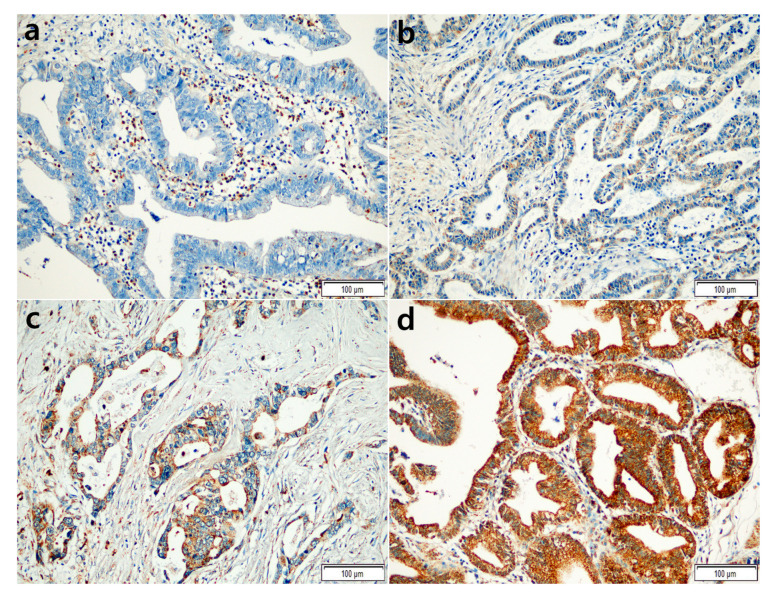
Representative images of immunohistochemistry (IHC) staining with CADM4 in gallbladder cancer (GBC) (×200). Negative (**a**), weak (**b**), moderate (**c**), and strong (**d**) cytoplasmic expression.

CADM4 expression was assessed using the immunoreactivity score (IRS), as previously described [20]. Cytoplasmic staining of tumor cells was considered for CADM4 expression and semi-quantitatively evaluated independently by two pathologists (Bang, S. and Paik, S.) who were blinded to the clinical information. The intensity of IHC staining was classified as 0: negative, 1: weak, 2: moderate, and 3: strong. The proportion of IHC staining was graded as 0 (0%), 1 (1–25%), 2 (26–50%), 3 (51–75%), and 4 (>75%). The IRS was then calculated by multiplying the proportion of positive cells by the staining intensity, ranging from 0–12. Representative microphotographs of IHC staining are shown in Figure 4. A receiver operating characteristics (ROC) curve analysis was performed using overall survival (OS), and the optimal cutoff value determined as the point maximizing the Youden index. Based on the CADM4 expression level, the cases were divided into low and high expression groups (IRS ≤ 4 and IRS > 4, respectively).

### 4.5. Assessment of HER2 Status

Silver in situ hybridization (SISH) was performed on TMA slides to confirm HER2 amplification. Automated staining was performed using INFORM HER2 Dual ISH DNA probe cocktail (Roche, Basel, Switzerland), according to the manufacturer’s protocol. The numbers of black (HER2) signals and red (CEP17) signals were counted under light microscopy. Then, as previously described by Sung et al. [19], cases with a HER2/CEP17 ratio ≥2 were regarded as positive for HER2 amplification.

### 4.6. Statistical Analyses

All statistical analyses were performed using SPSS software version 25.0 (IBM, Armonk, NY, USA). The correlation between CADM4 expression and clinicopathological characteristics was analyzed using Pearson’s chi-square or Fisher’s exact test. To determine the effect of CADM4 expression on OS and recurrence-free survival (RFS), Kaplan–Meier analysis with the log-rank test was used. The Cox proportional hazards model was used to identify the prognostic factors of GBC patients. A two-tailed *p*-value of < 0.05 was considered statistically significant.

### 4.7. Pathway Enrichment Analysis

To obtain gene expression data in GBC by next-generation sequencing, GSE139682 (https://www.ncbi.nlm.nih.gov/geo/, accessed on 1 April 2023 ) deposited in the Gene Expression Omnibus (GEO) database was utilized. GSE139682 included the gene expression profiles of 10 human GBC samples, and we selected gene candidates showing significant expression differences between the low CADM4 expression and the high CADM4 expression groups using the GEO2R tool (https://www.ncbi.nlm.nih.gov/geo/geo2r/, accessed on 2 April 2023). We derived the top 250 candidates and considered 220 genes that met the cutoff value (log2FoldChange ≥ 1 or log2FoldChange ≤ −1, *p*-value < 0.05) as differentially expressed genes (DEGs). The Database for Annotation, Visualization and Integrated Discovery (DAVID) version 6.8 (https://david.ncifcrf.gov/home.jsp, accessed on 1 April 2023) was used as a functional annotation tool to perform Gene Ontology (GO) and Kyoto Encyclopedia of Genes and Genomes (KEGG) pathway enrichment analyses [23]. *p*-values < 0.05 were considered to be statistically significant.

## Figures and Tables

**Table 1:** Baseline characteristics of patients with gallbladder cancer (n = 100).

Characteristics	n (%)
Age, median (range, years)	65 (28–90)
Sex	
Female	51 (51.0%)
Male	49 (49.0%)
Tumor size, mean (range, cm) *	4.5 (0.2–10.0)
Location	
Neck	12 (12.0%)
Body	48 (48.0%)
Fundus	22 (22.0%)
Neck to body	5 (5.0%)
Body to fundus	5 (5.0%)
Neck to fundus	4 (4.0%)
Whole gallbladder	4 (4.0%)
Histological type	
Adenocarcinoma	89 (89.0%)
Adenocarcinoma with neuroendocrine differentiation	2 (2.0%)
Adenocarcinoma with sarcomatoid differentiation	3 (3.0%)
Mixed adenocarcinoma	2 (2.0%)
Adenosquamous carcinoma	4 (4.0%)
Histological grade	
G1 (well differentiated)	22 (22.0%)
G2 (moderately differentiated)	57 (57.0%)
G3 (poorly differentiated)	21 (21.0%)
Lymphovascular invasion	
Present	50 (50.0%)
Not identified	50 (50.0%)
Perineural invasion	
Present	32 (32.0%)
Not identified	68 (68.0%)
T category	
pT1a	8 (8.0%)
pT1b	9 (9.0%)
pT2	49 (49.0%)
pT3	31 (31.0%)
pT4	3 (3.0 %)
N category	
pNx	15 (15.0%)
pN0	49 (49.0%)
pN1	33 (33.0%)
pN2	3 (3.0%)
TNM stage (AJCC 8th edition)	
I	17 (17.0%)
II	34 (34.0%)
IIIA	13 (13.0%)
IIIB	31 (31.0%)
IVA	2 (2.0%)
IVB	3 (3.0%)
HER2 status	
No amplification	91 (91.0%)
Amplification	9 (9.0%)

* Tumor size, 8 cases missed. AJCC, American Joint Committee on Cancer; HER2, epidermal growth factor receptor 2.

**Table 2:** Correlations between CADM4 expression and clinicopathological characteristics in patients with gallbladder cancer (n = 100).

Variables	CADM4 Expression		*p*-Value
	Low Expression (%) (n = 28)	High Expression (%) (n = 72)	
Age			0.914
<65 years	12 (28.6%)	30 (71.4%)	
≥65 years	16 (27.6%)	42 (72.4%)	
Sex			0.748
Female	15 (29.4%)	36 (70.6%)	
Male	13 (26.5%)	36 (73.5%)	
Histological grade			0.948
Grade 1 or 2	22 (27.8%)	57 (72.2%)	
Grade 3	6 (28.6%)	15 (71.4%)	
Lymphovascular invasion			1.000
Not identified	14 (28.0%)	36 (72.0%)	
Present	14 (28.0%)	36 (72.0%)	
Perineural invasion			0.620
Not identified	18 (26.5%)	50 (73.5%)	
Present	10 (31.3%)	22 (68.8%)	
T category			0.010
pT1 or pT2	13 (19.7%)	53 (80.3%)	
pT3 or pT4	15 (44.1%)	19 (55.9%)	
N category			0.373
pNx or pN0	16 (25.0%)	48 (75.0%)	
pN1 or pN2	12 (33.3%)	24 (66.7%)	
TNM stage (AJCC 8th edition)			0.019
I or II	9 (17.6%)	42 (82.4%)	
III or IV	19 (38.8%)	30 (61.2%)	
HER2 status			0.262 *
No amplification	24 (26.4%)	67 (73.6%)	
Amplification	4 (44.4%)	5 (55.6%)	

* Fisher’s exact test. AJCC, American Joint Committee on Cancer; HER2, epidermal growth factor receptor 2.

**Table 3:** Univariate and multivariate Cox regression analyses in patients with gallbladder cancer (n = 100).

OS						
Variables	Univariate Analysis			Multivariate Analysis		
	HR	95% CI	*p-*Value	HR	95% CI	*p-*Value
CADM4 expression (high vs. low)	2.654	1.440–4.892	0.002	2.199	1.182–4.091	0.013
Age group (<65 years vs. ≥65 years)	1.505	0.805–2.816	0.201			
Histological grade (grade 1 or 2 vs. grade 3)	1.604	0.820–3.134	0.167			
T category (pT1 or pT2 vs. pT3 or pT4)	2.889	1.570–5.313	0.001			
N category (pNx or pN0 vs. pN1 or pN2)	2.628	1.427–4.838	0.002			
TNM stage (AJCC 8th edition) (I or II vs. III or IV)	3.475	1.775–6.803	< 0.001	2.267	1.040–4.944	0.040
Lymphovascular invasion (not identified vs. present)	2.507	1.337–4.700	0.004	1.424	0.682–2.976	0.347
Perineural invasion (not identified vs. present)	2.493	1.348–4.613	0.004	1.419	0.688–2.925	0.343
HER2 status (no amplification or amplification)	0.996	0.391–2.539	0.994			
**RFS**						
**Variables**	**Univariate Analysis**			**Multivariate Analysis**		
	**HR**	**95% CI**	***p-*** **Value**	**HR**	**95% CI**	***p-*** **Value**
CADM4 expression (high vs. low)	2.181	1.114–4.271	0.023	1.923	0.978–3.782	0.058
Age group (<65 years vs. ≥65 years)	0.691	0.359–1.331	0.269			
Histological grade (grade 1 or 2 vs. grade 3)	1.451	0.682–3.088	0.334			
T category (pT1 or pT2 vs. pT3 or pT4)	5.401	2.690–10.845	< 0.001			
N category (pNx or pN0 vs. pN1 or pN2)	3.141	1.613–6.116	0.001			
TNM stage (AJCC 8th edition) (I or II vs. III or IV)	9.230	3.577–23.813	< 0.001	5.726	2.077–15.788	0.001
Lymphovascular invasion (not identified vs. present)	4.945	2.240–10.919	< 0.001	2.398	0.977–5.882	0.056
Perineural invasion (not identified vs. present)	3.059	1.580–5.923	0.001	1.234	0.589–2.586	0.577
HER2 status (no amplification or amplification)	0.466	0.112–1.942	0.294			

OS, overall survival; RFS, recurrence-free survival; CADM4, cell adhesion molecule 4; AJCC, American Joint Committee on Cancer; HER2, epidermal growth factor receptor 2; HR, hazard ratio; 95% CI, 95% confidence interval.

## Data Availability

Publicly available datasets were analyzed in this study. This data can be found here: (https://www.ncbi.nlm.nih.gov/geo/query/acc.cgi?acc=GSE139682, accessed on 10 March 2023).

## Supplementary Materials

The following supporting information can be downloaded at: https://www.mdpi.com/article/10.3390/ijms24086898/s1.
